# Die extrakorporale Stoßwellentherapie als Therapiealternative bei posttraumatischer verzögerter Knochenheilung

**DOI:** 10.1007/s00113-022-01225-5

**Published:** 2022-08-26

**Authors:** Sebastian Hempe, Dan Bieler, Grit Braunegger, Thomas Schilling, Stephan Waldeck, Erwin Kollig

**Affiliations:** 1 Klinik für Unfallchirurgie und Orthopädie, Wiederherstellungschirurgie, Handchirurgie, Verbrennungsmedizin, Bundeswehrzentralkrankenhaus Koblenz, Rübenacher Str. 170, 56072 Koblenz, Deutschland; 2 Klinik für Orthopädie und Unfallchirurgie, Heinrich-Heine-Universität Düsseldorf, Düsseldorf, Deutschland; 3 Klinik für Nuklearmedizin, Bundeswehrzentralkrankenhaus Koblenz, Koblenz, Deutschland; 4 Klinik für Radiologie und Neuroradiologie, Bundeswehrzentralkrankenhaus Koblenz, Koblenz, Deutschland

**Keywords:** Konservative Therapie, ESWT, Pseudarthrose, Knochenheilungsstörung, Frakturheilung, Conservative treatment, ESWT, Pseudarthrosis, Impaired bone healing, Fracture healing

## Abstract

**Hintergrund:**

Posttraumatische Knochenheilungsstörungen stellen eine relevante Komplikation von Frakturen dar. Die operative Revision hat sich als Standardtherapie etabliert. Als alternatives, nichtoperatives Behandlungsverfahren kann die extrakorporale Stoßwellentherapie (ESWT) die Möglichkeit bieten, die potenziellen Komplikationen eines operativen Vorgehens zu vermeiden.

**Ziel der Arbeit:**

Die Ergebnisse einer eigenen Fallserie sollen dargestellt und mit der aktuellen Literatur verglichen werden.

**Material und Methoden:**

Im Zeitraum von 2007 bis 2016 wurden 97 Patienten mit posttraumatischer Knochenheilungsstörung alternativ zu einer Revisionsoperation mittels ESWT behandelt. Klinische und demografische Parameter dieses Kollektivs wurden erhoben und ausgewertet. Primärer Endpunkt war die knöcherne Ausheilung. Verschiedene Faktoren wurden hinsichtlich ihres Einflusses auf die Frakturkonsolidierung untersucht.

**Ergebnisse:**

Nach ESWT konnte eine Konsolidierungsrate von 60,8 % erzielt werden. Eine präinterventionelle Diastase ≥ 5 mm, eine initiale Dislokation > ½ Schaftbreite, aktiver Nikotinkonsum sowie ein Zeitraum von der Fraktur bis zur ESWT > 6 Monate wurden als signifikant negative Einflussfaktoren identifiziert. Es traten keine relevanten Komplikationen auf.

**Schlussfolgerung:**

Die ESWT ist eine sichere und vielversprechende Therapiealternative bei posttraumatisch verzögerter Knochenheilung. Unter Berücksichtigung von vorab zu identifizierenden Risikofaktoren kann ihre Erfolgsrate gesteigert werden.

**Graphic abstract:**

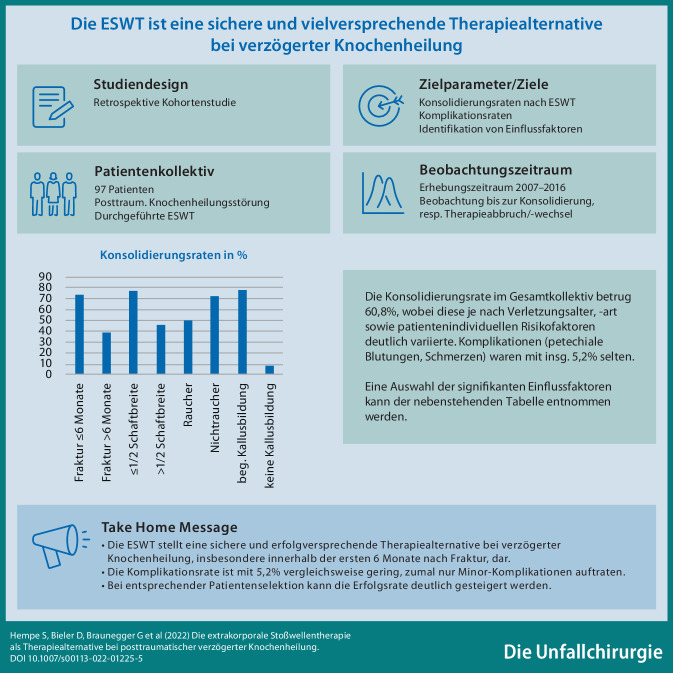

## Abstract (en)

**Background:**

Impaired posttraumatic bone healing is a relevant complication of fractures. Usually, the standard treatment is surgical revision. For about 30 years extracorporeal shockwave therapy (ESWT) has emerged as an alternative treatment option with similar consolidation rates but less complications.

**Objective:**

This article aims to present our data in context to the current literature

**Material and methods:**

From 2007 to 2016 a total of 97 patients diagnosed with impaired posttraumatic bone healing were treated with ESWT. Clinical and demographic data of this population were retrieved and analyzed retrospectively.

**Results:**

The general consolidation rate was 60.8%. Multiple variables were analyzed. A preinterventional bone gap ≥ 5 mm, initial dislocation > ½ of the bone shaft, nicotine consumption and a long time span from fracture to ESWT (> 6 months) were found as factors which significantly impair bone healing after ESWT.

**Conclusion:**

ESWT is a safe and promising alternative treatment option for delayed unions. Regarding risk factors of a poor outcome may be identified before and increase the rate of success.

Posttraumatische Knochenheilungsstörungen stellen eine relevante Komplikation in der Behandlung von Frakturen dar, insbesondere der langen Röhrenknochen. Die oft langwierigen Heilungsverläufe mit Revisionsoperationen bedeuten für Betroffene eine belastende Situation und bergen das Risiko der typischen Komplikationen eines operativen Eingriffs. Weiterhin relativieren relevante, individuelle Komorbiditäten die Indikation für einen operativen Eingriff. Mit der extrakorporalen Stoßwellentherapie (ESWT) wurde eine komplikationsarme, nichtinvasive Therapiealternative zum operativen Standard etabliert. In dieser Studie soll die Effektivität der ESWT in der Behandlung postoperativer Knochenheilungsstörungen beleuchtet werden.

Postoperative Knochenheilungsstörungen und Pseudarthrosen treten in ca. 5–10 % [9] aller Frakturen auf und stellen bei einer Frakturinzidenz von 1014/100.000 erwachsenen Einwohnern in Deutschland [22] eine klinisch wie auch volkswirtschaftlich bedeutsame Komplikation dar. Die Wahrscheinlichkeit für das Auftreten im Bereich der unteren Extremität, insbesondere an der Tibia, wird in einzelnen Studienkollektiven mit bis zu 45 % höher angegeben [13].

Abhängig von der Dauer des Heilungsverlaufs wird im deutschsprachigen Raum zwischen der verzögerten Knochenheilung bzw. „delayed-union“ (DU) und der Pseudarthrose (PA) bzw. „non-union“ (NU) unterschieden. Erstere wird durch das Ausbleiben der knöchernen Konsolidierung über einen Zeitraum von mehr als 3 Monaten hinaus definiert, wohingegen das Ausbleiben der knöchernen Konsolidierung nach einem Zeitraum von mehr als 6 Monaten mit fehlender Aussicht auf Konsolidierung die PA davon abgrenzt. Im Gegensatz hierzu wird im englischsprachigen Raum eine Pseudarthrose erst angenommen, wenn die Ausheilung über mehr als 9 Monate ausbleibt und über einen Zeitraum von mind. 3 Monaten kein Progress in der knöchernen Durchbauung festgestellt werden kann [15, 24]. Ein exemplarischer Röntgenbefund für eine DU ist in Abb. 1 dargestellt.

**Figure 1:**
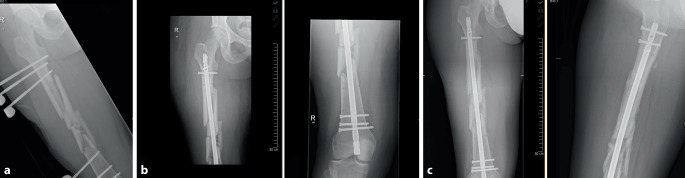

Die Gründe für die Entstehung einer postoperativen Knochenheilungsstörung sind vielfältig und können in 4 Gruppen gegliedert werden:

- mechanisch (z. B. insuffiziente Osteosynthese),
- biologisch (bspw. mangelhafte Durchblutung),
- defektbedingt und
- infektbedingt [24].

Als Diagnosetool zur Unterscheidung der Genese hat sich zusätzlich zu der ursprünglich durch Weber und Cech [30] beschriebenen Einteilung in vitale und avitale Pseudarthrosen anhand morphologischer Kriterien (hypertroph, oligotroph, atroph etc.) das „Non-Union Scoring System“ (NUSS) nach Calori et al. [8] durchgesetzt. Es erfasst Faktoren wie Frakturmorphologie, erfolgte operative Therapie, aber auch patientenindividuelle Risikofaktoren, wie Vorerkrankungen und etwaigen Nikotinabusus. Es soll der Differenzierung zwischen mechanisch und biologisch bedingter Knochenheilungsstörung dienen und damit bei der Therapieentscheidung unterstützen. Bis dato gilt die operative Revision mit Anfrischen der Frakturenden, Optimierung der Osteosynthese und ggf. Einbringen von autologer Spongiosa bzw. die Überbrückung einer Defektstrecke mit z. B. Kallusdistraktion als Standardtherapie [12, 16]. Hierbei bildet das 2007 eingeführte und international anerkannte Diamond-Konzept nach Giannoudis et al. [1, 14] einen Leitfaden für die Therapieplanung, indem es Kriterien definiert, welche eine entscheidende Rolle in der Knochenheilung und damit auch in der Therapie posttraumatischer Knochenheilungsstörungen spielen. Die in Tab. 1 dargestellten Faktoren sollten demnach im Rahmen der operativen Therapie berücksichtigt werden.

**Table 1:** 

Zu optimierender Faktor	Erzielbar durch/enthalten in
Mechanische Stabilität	Optimierung der Osteosynthese
Vorhandensein osteogener Zellen (z. B. mesenchymale Stammzellen (MSC))	Frakturhämatom, autologe Knochentransplantation
Vorhandensein osteokonduktiver Strukturen	Z. B. autologe Knochentransplantation
Optimale Vaskularisation	Z. B. Optimierung patientenindividueller Risikofaktoren, Anfrischen der Frakturenden
Vorhandensein von Wachstumsfaktoren	Frakturhämatom, thrombozytenreiches Plasma, Wachstumsfaktorpräparate

In der Literatur werden die Heilungsraten bei Beachtung aller oben genannten Kriterien im Sinne einer „Polytherapie“ mit 80–98 % angegeben [1, 19]. Werden nicht alle Faktoren bedacht, sind Heilungsraten von 44–90 % beschrieben [1].

Bereits seit den 1990er-Jahren wurde mit der extrakorporalen Stoßwellentherapie (ESWT) eine gering invasive Therapiealternative in der Behandlung posttraumatischer Knochenheilungsstörungen eingeführt [28], die Heilungsraten bis zu 70–94 % [2, 15] und vernachlässigbare Komplikationsraten [23] bietet. Als Wirkmechanismus wurden zunächst nur die direkten und histologisch nachweisbaren Wirkungen auf den Knochen z. B. durch Mikrofrakturierung angenommen [27]. Nach heutigem Verständnis handelt es sich jedoch um eine komplexe biomechanische Wechselwirkung der Stoßwelle mit dem exponierten Gewebe. Die Hypothese der „Mechanotransduktion“ [16, 23] geht davon aus, dass die Stoßwelle Druck- und Scherkräfte hervorruft, die wiederum diverse Signalkaskaden auslöst und zu einer vermehrten Expression von Enzymen und Wachstumsfaktoren sowie einer vermehrten Angioneogenese führt. Hierzu gehören u. a. die endotheliale Stickstoffmonoxidsynthase (eNOS), der „vascular endothelial growth factor“ (VEGF), das „proliferating cell nuclear antigen“ (PCNA) und „bone morphogenetic protein 2“ (BMP2). [4, 29]. Im Tiermodell konnte aufgezeigt werden, dass diese Kaskade eine vermehrte Migration und Differenzierung mesenchymaler Stammzellen auslöst und die enchondrale Ossifikation fördert [18]. Das bildmorphologische Korrelat einer Ausheilung nach ESWT ist in Abb. 2 dargestellt.

**Figure 2:**
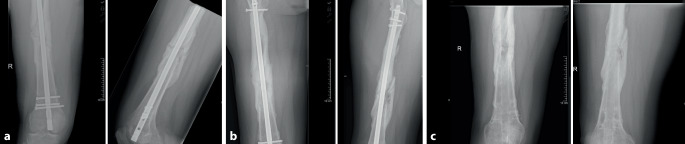

Von der hier angewandten hochenergetischen, fokussierten ESWT ist mit der radialen Druckwellentherapie ein weiteres Therapieverfahren abzugrenzen, dessen Wirkung auf dem Mechanismus der „Mechanotransduktion“ zu beruhen scheint. Wesentlicher Unterschied ist die sich hierbei radial ausbreitende Energie mit einer insgesamt deutlich niedrigeren Druckamplitude und, damit verbunden, niedrigeren Eindringtiefe in das Gewebe. Indikationen für die radiale Druckwellentherapie sind dementsprechend eher oberflächliche Gewebeveränderungen oder Schmerzzustände, wohingegen die hochenergetische, fokussierte ESWT für Behandlungen in der Tiefe angewendet werden kann [26]. Ziel dieser Studie ist es, die klinische Effektivität der ESWT auch in Anbetracht diverser Variablen anhand des eigenen Patientengutes, welches mit dem Modulith SLK (Fa. STORZ MEDICAL, Tägerwilen, Schweiz) behandelt wurde, zu untersuchen.

## Material und Methode

### Ethische Regelungen

Bei dieser rein retrospektiven Datenerhebung, aus standardmäßig erfassten und bereits vorliegenden Daten, wurde auf die Einreichung eines Ethikantrags im Einklang mit den aktuellen, in Rheinland-Pfalz geltenden Richtlinien verzichtet.

### Datenerhebung und Analyse

Im Rahmen einer retrospektiven Erhebung wurden via OPS(Operationen- und Procedurenschlüssel)- (8–115) und ICD(International Classification of Diseases)-Code-Suche (M84.1; M84.2) im Krankenhausinformationssystem sämtliche Patienten identifiziert, die im Zeitraum von 2007 bis 2016 aufgrund einer posttraumatischen Knochenheilungsstörung im Bereich der Röhrenknochen mittels hochenergetischer ESWT behandelt wurden. Die Ein- und Ausschlusskriterien können Tab. 2 entnommen werden. Demografische und klinische Patientendaten wurden anhand der vorhandenen Falldokumentation erhoben und verarbeitet. Als primäre Zielgröße wurde die radiologisch mittels biplanarer Röntgenbildgebung gesicherte knöcherne Konsolidierung definiert. Anschließend wurde der Einfluss verschiedener Variablen auf die Konsolidierung resp. Nichtkonsolidierung analysiert.

**Table 2:** 

Einschlusskriterien	Ausschlusskriterien
Posttraumatische Knochenheilungsstörung	Knocheninfektion
Lokalisation an Röhrenknochen	Lokalisation an anderen Knochen
Operative oder konservative Frakturbehandlung	ESWT nach Arthrodese
Nachbehandlung in domo	

Die statistische Auswertung erfolgte mittels Chi-Quadrat-Test, dem Exakten Test nach Fischer sowie der logistischen Regression. Das Signifikanzniveau wurde bei allen Tests auf *p* = 0,05 festgelegt. Das verwendete Statistikprogramm war SPSS® Version 26 für Windows (International Business Machines Corporation, IBM). Patienten, bei denen aufgrund des retrospektiven Settings einzelne Daten nicht erhoben werden konnten, wurden im Sinne des paarweisen Fallausschlusses aus den Einzelanalysen exkludiert.

### ESWT

**Figure 3:**
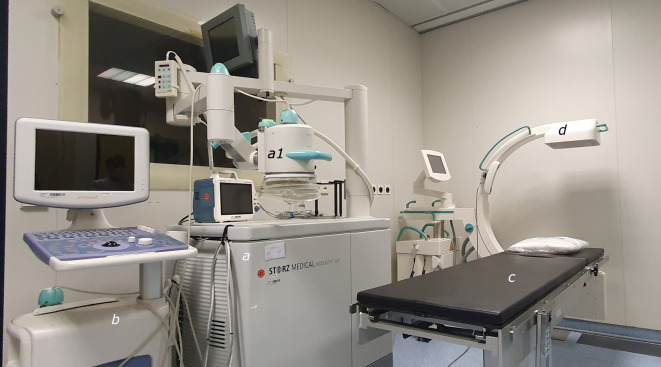

Die Indikationsstellung zur hochenergetischen, fokussierten ESWT erfolgte in der interdisziplinären unfallchirurgisch-radiologischen Fallbesprechung. Die Therapie wurde mit dem Modulith® SLK der Fa. STORZ MEDICAL (Abb. 3), in der Regel mit einer einmaligen Stoßwellenapplikation, durchgeführt. Die Therapievariablen in Abhängigkeit von der Lokalisation können Tab. 3 entnommen werden.

**Table 3:** 

	Obere Extremität (exkl. Hand)	Untere Extremität (exkl. Fuß)	Hand/Fuß
Energieflussdichte (mJ/mm^2^)	0,853 ± 0,209	0,834 ± 0,256	0,580 ± 0,132
*Anzahl der Therapiesitzungen*			
1	14	53	10
2	1	15	2
3	0	2	0
*Anzahl der Stoßwellen*			
2000–4000	5	5	4
4000–8000	7	26	7
8000–10.000	0	11	1
> 10.000	3	28	0
*Analgesie/Anästhesie*			
Systemisch	11	45	4
Lokal	1	10	5

## Ergebnisse

Insgesamt wurden *n* = 79 (81,4 %) männliche und *n* = 18 (18,6 %) weibliche Patienten in die Studie eingeschlossen. Die Altersverteilung reichte von 20 bis 79 Jahren mit *n* = 51 Patienten (52,5 %) im Altersbereich von 30 bis 49 Jahren. Tab. 4 bietet eine Übersicht über das eingeschlossene Gesamtkollektiv.

**Table 4:** 

*Gesamt*	97
*Weiblich*	18
*Alter*	
< 20 Jahre	9
– 20–29 Jahre	18
– 30–39 Jahre	24
– 40–49 Jahre	27
– 50–59 Jahre	12
≥ 60 Jahre	7
*Vorerkrankungen*	
– Diabetes mellitus	5
– Durchblutungsstörung	3
– Knochenstoffwechselstörung	4
– Neuropathie	13
– Rheumatische Erkrankung	3
*Nikotinabusus*	44
*Betroffener Knochen*	
– Femur	26
– Tibia/Fibula	44
– Humerus	3
– Radius/Ulna	12
– Metacarpalia/-tarsalia, Phalangen	12
*Primär operative Therapie*	95
*Kallusdistraktion*	7

Im vorliegenden Kollektiv zeigte sich eine Konsolidierungsrate nach ESWT von 60,8 % (*n* = 59) bei einer Komplikationsrate von 5,2 % (*n* = 5), wobei hier lediglich das Auftreten von Hämatomen, lokalen Schmerzen und petechialen Blutungen festgestellt wurde. Schwerwiegende Komplikationen traten nicht auf. Die Konsolidierungsraten bei Patienten mit offener (*n* = 38) und geschlossener (*n* = 59) Fraktur unterschieden sich nicht signifikant (*p* = 0,185). Auch eine primäre Gelenkbeteiligung (*p* = 0,368) hatte keinen signifikanten Einfluss auf die Konsolidierungsrate. Hingegen konnten das initiale Dislokationsausmaß (*p* = 0,002) und die präinterventionelle Diastase zwischen den Frakturenden (*p* = 0,001) als signifikante Einflussfaktoren identifiziert werden. Patienten, bei denen zuvor ein operativer Verfahrenswechsel erfolgte (*n* = 32), wiesen eine signifikant niedrigere Konsolidierungsrate auf (*p* = 0,016).

Die Zeitspanne vom Zeitpunkt der Fraktur bis zur ESWT lag bei 7,87 ± 8,4 Monaten und zeigt in der binären logistischen Regression ebenfalls einen signifikanten Einfluss auf die Konsolidierungsrate (*p* = 0,001). In der durchgeführten Subgruppenanalyse konnte hier eine signifikant höhere Konsolidierungsrate bei Durchführung der ESWT innerhalb der ersten 6 Monaten nach Fraktur vs. > 6 Monate nachgewiesen werden (*p* = 0,001). Patienten mit einem Nikotinabusus wiesen eine signifikant niedrigere Konsolidierungsrate auf (*p* = 0,029). Auch zeigte sich bei einer zum Zeitpunkt der ESWT bereits eingesetzten, aber unvollständigen Kallusbildung (hyper- oder oligotroph) eine höhere Durchbauungsrate (*p* < 0,001). Patienten, bei denen die Notwendigkeit eines aufwendigen Weichteilmanagements (Maßnahmen der plastisch-rekonstruktiven Leiter, welche über die einfache Wundnaht hinaus gehen) bestand, wiesen signifikant schlechtere Konsolidierungsraten auf (*p* = 0,021).

Eine Übersicht der betrachteten Variablen ist in Tab. 5 aufgelistet.

**Table 5:** 

Merkmalsausprägung	Konsolidierung	Konsolidierungsrate (in %)	*p*-Wert
Männlich vs. weiblich	47/79 vs. 12/18	59,5 vs. 66,77	0,574
Offene vs. geschlossene Fraktur	20/38 vs. 39/59	52,6 vs. 66,1	0,185
Gelenkbeteiligung vs. keine Gelenkbeteiligung	38/59 vs. 21/38	66,4 vs. 55,3	0,368
Primäre Dislokation ≤ ½ Schaftbreite vs. > ½ Schaftbreite	34/44 vs. 24/52	77,3 vs. 46,2	*0,002*
Diastase < 5 mm vs. ≥ 5 mm	45/66 vs. 7/24	68,2 vs. 29,2	*0,001*
Kein Verfahrenswechsel vs. Verfahrenswechsel	45/65 vs. 14/32	69,2 vs. 43,8	*0,016*
Aufwendiges vs. einfaches Weichteilmanagement	12/28 vs. 47/69	42,9 vs. 68,1	*0,021*
Zeitabstand von Fraktur zu ESWT ≤ 6 Monate vs. > 6 Monate	45/61 vs. 14/36	73,8 vs. 38,9	*0,001*
Nikotinabusus vs. Nikotinabstinenz	22/44 vs. 36/50	50 vs. 72	*0,029*
Begonnene vs. keine Kallusbildung vor ESWT	57/73 vs. 2/24	78,1 vs. 8,3	*<* *0,001*

Im vorliegenden Patientenkollektiv bestanden insgesamt nur wenige Vorerkrankungen zum Zeitpunkt der ESWT. Diese zeigten, soweit bei geringer Fallzahl auswertbar, keinen signifikanten Einfluss auf die Konsolidierung.

## Diskussion

Die extrakorporale Stoßwellentherapie stellt bereits seit den 1990er-Jahren eine effektive Alternative in der Therapie von postoperativen Knochenheilungsstörungen dar [20, 27, 28]. Die publizierten Konsolidierungsraten von 29 % bis zu > 90 % [3, 11, 12] unterscheiden sich zwar teilweise deutlich, bestärken aber insgesamt die Annahme einer hohen Wirksamkeit dieser Therapieoption bei klarer Indikationsstellung. Zu diesem Schluss kommen auch Willems et al. [31] in ihrem insg. 28 Studien umfassenden systematischen Review, in dem allerdings auch kritisch auf die Qualität der ausgewerteten Daten wegen fehlender Prospektivität und Randomisierung hingewiesen wird. Die in der vorliegenden Studie erhobene Konsolidierungsrate von 60,8 % ist mit diesen bisher veröffentlichten Daten vergleichbar. Diese sind allerdings nur eingeschränkt miteinander vergleichbar, da sie sich hinsichtlich der Lokalisation der behandelten Knochen, ihrer Einschlusskriterien und Therapieprotokolle unterscheiden [23]. So wiesen bereits Everding et al. [11] in ihrer Analyse darauf hin, dass häufig unterschiedliche Methoden zur Generierung der Stoßwellen, mit jeweils eigenen Besonderheiten und unterschiedlichen Energieflussdichten, angewendet wurden. Ferner beschreiben u. a. Elster et al. [10] und Haffner et al. [16] Kollektive mit Pseudarthrosen ausschließlich im Bereich der Tibia, mit Heilungsraten von 80,2 %, resp. 88,5 %, wohingegen Everding et al. [11] ein Kollektiv mit Pseudarthrosen in unterschiedlichen Lokalisationen und einer Konsolidierungsrate von 73 % darstellten, jedoch Patienten mit einer Diastase > 5 mm ausschlossen. In der vorliegenden Untersuchung wurden Patienten mit Knochenheilungsstörungen an unterschiedlichen Lokalisationen und unter Einschluss solcher mit größerer Diastase (≥ 5 mm) einbezogen. Letztere wiesen eine signifikant schlechtere Heilungsrate (29 %, *p* = 0,001) auf. Dieses Ergebnis ist mit dem von Schaden et al. [23] vergleichbar und bestärkt den von Everding et al. [12] definierten Therapiealgorithmus, der eine ESWT-Durchführung insbesondere bei Spaltmaßen < 5 mm empfiehlt. Die Daten der vorliegenden Studie weisen darauf hin, dass der späte Beginn einer ESWT grundsätzlich mit einem schlechten Ansprechen des Knochengewebes auf eine Stoßwellentherapie verbunden sein könnte und Patienten mit einem Therapiebeginn mehr als 6 Monate nach der Fraktur eine signifikant niedrigere Heilungsrate aufweisen (*p* = 0,001). Dies deckt sich mit Beobachtungen von Haffner et al. [16], welche eine signifikant kürzere Zeitdauer von der letzten operativen Intervention bis zur ESWT in der Subgruppe von Patienten mit vollständiger Konsolidierung nachweisen konnten (*p* < 0,0001), wobei in deren Patientengut ausschließlich manifeste Pseudarthrosen ausgewertet wurden, d. h. solche mit einer Krankheitsgeschichte von mehr als 6 Monaten nach Trauma. Die Zeitdauer vom Trauma bis hin zur ESWT zeigte sich in der genannten Studie knapp nichtsignifikant (*p* = 0,057), was am ehesten mit dem kleinen Patientenkollektiv erklärbar ist. In einer Subgruppenanalyse im Kollektiv mit einem Zeitintervall vom Trauma bis zur ESWT-Durchführung ≤ 6 Monate konnte in der vorliegenden Erhebung kein signifikanter Unterschied hinsichtlich der Konsolidierungsraten festgestellt werden. Die Beobachtung, dass eine höhere Konsolidierungsrate bei bereits beginnender Kallusbildung (*p* < 0,001) zu verzeichnen war, kann als indirekter Hinweis darauf gewertet werten, dass die EWST auf eine biologische Mindestaktivität in der Frakturzone angewiesen ist, um ihre Wirkung z. B. hinsichtlich Neovaskularisation und Mediatorenfreisetzung zu entfalten, und könnte erklären, wieso sie eine schlechtere Wirksamkeit bei atrophen Knochenheilungsstörungen und Pseudarthrosen aufweist. In der bereits zitierten Studie von Everding et al. [11] fand sich dieser Zusammenhang interessanterweise nicht. Auch wenn sich die Heilungsraten nach ESWT in Abhängigkeit vom Patientengeschlecht nicht signifikant unterschieden (*p* = 0,547), so ist es doch auffällig, dass das vorliegende Patientenkollektiv deutlich mehr männliche als weibliche Patienten bzw. Patientinnen beinhaltete (81,4 % vs. 18,6 %). Ähnliche Verhältnisse finden sich in den Studien von Elster et al. (70,9 % vs. 29,1 %) [10], Everding et al. (71,8 % vs. 28,2 %) [11] sowie Haffner et al. (76,1 % vs. 23,9 %) [16]. Dies kann als Indiz dafür angesehen werden, dass posttraumatische Knochenheilungsstörungen beim männlichen Geschlecht häufiger aufzutreten scheinen, bzw. dass Männer häufiger diese Verletzungen erleiden.

Wie bereits in diversen Studien dargestellt [17, 25], konnte auch im vorliegenden Datensatz nachgewiesen werden, dass sich ein Nikotinabusus signifikant negativ auf die Knochenheilung auswirkt (*p* = 0,029). Weiterhin konnte in dieser Studie ein signifikanter Einfluss des initialen Dislokationsgrads (> 1/2 Schaftbreite, *p* = 0,002) sowie der Notwendigkeit eines komplexen Weichteilmanagements (*p* = 0,021) auf das Ergebnis einer ESWT-Anwendung festgestellt werden. Dies überrascht nicht, da größere Weichteilverletzungen sowie die Frakturmorphologie bekannte Risikofaktoren in der Entstehung von Pseudarthrosen darstellen [7, 9]. Hier ist es nicht erklärbar, warum im vorliegenden Patientenkollektiv kein signifikanter Unterschied bei den Konsolidierungsraten von initial offenen gegenüber geschlossenen Frakturen vorlag (*p* = 0,185). Ein möglicher Erklärungsansatz wäre hier, dass offene Frakturen nicht immer mit einer ausgedehnten Weichteilschädigung einhergehen müssen. Ähnliche Tendenzen, jedoch ebenfalls ohne Signifikanz, finden sich in den bereits zitierten Publikationen von Elster et al. (75,8 % vs. 82,7 %, *p* = 0,34) [10] und Haffner et al. (80 % vs. 96,3 %, *p* = 0,094) [16]. Auch wenn in der Literatur ein negativer Einfluss von Co-Morbiditäten wie Diabetes mellitus auf die Knochenheilung beschrieben wird [9, 12], konnte in unserem Kollektiv kein signifikanter Einfluss nachgewiesen. Dies könnte mit der kleinen Stichprobengröße zusammenhängen.

Die ESWT stellt eine Behandlungsoption von Pseudarthrosen dar, die im Vergleich zur operativen Revision wenig invasiv, ambulant und kostengünstig bzw. ressourcenschonend realisierbar ist. Bereits 2017 zeigten Everding et al. [11] anhand einer Beispielrechnung an einem Kollektiv von 39 Patienten, dass dieses Verfahren mit weniger als einem Viertel der Kosten der operativen Therapiealternative um ein Vielfaches günstiger ist. Die unverändert schlechte Datenlage mit dem Fehlen prospektiver, randomisierter Untersuchungen ist der Grund, weshalb sich dieses Verfahren noch keinen Platz im Leistungskatalog der gesetzlichen Krankenkassen sichern konnte. Während das Prinzip der Stoßwellentherapie an sich z. B. in der Therapie der Plantarfasziitis zugelassen ist [5], kann die ESWT zur Behandlung von Knochenheilungsstörungen bisher nur bei Unfallverletzten und Selbstzahlern kostendeckend angeboten werden. Die hieraus in der breiten Masse entstehende mangelnde Rentabilität hat in einzelnen Einrichtungen bereits zu einer kompletten Beendigung der ESWT-Behandlung von Knochenheilungsstörungen geführt [21].

## Schlussfolgerung

Insgesamt bleibt festzuhalten, dass die ESWT eine effektive Behandlungsalternative in der Therapie posttraumatischer Knochenheilungsstörungen, insbesondere im Falle der verzögerten Knochenheilung darstellt. In Anbetracht der im Vergleich zur operativen Vorgehensweise sehr niedrigen Rate an Komplikationen (bis zu 34 % [15] vs. 5,2 % in dieser Studie) bei vergleichbaren Konsolidierungsraten [6], sollte die ESWT Patienten mit posttraumatischen Knochenheilungsstörungen zumindest als Therapiealternative aufgezeigt werden. Aufgrund der häufigen Verweigerung einer Kostenübernahme seitens der Gesetzlichen Krankenversicherungen (GKV) ist vor ESWT-Durchführung grundsätzlich eine Kostenübernahmeerklärung beim Kostenträger einzuholen. Die bisher ablehnende Haltung der GKV gegenüber der ESWT kann nur durch mehr Evidenz, d. h. mit weiteren Studien mit prospektivem und randomisiertem Studiendesgin, geändert werden.

## Limitationen

Die in dieser Studie erhobenen Daten basieren auf einer retrospektiven Auswertung. Die Stichprobengröße erlaubt keine weitere Auswertung von Subgruppen, v. a. hinsichtlich des Einflusses von Begleiterkrankungen auf das Therapieergebnis.

## Fazit für die Praxis

- Posttraumatische Knochenheilungsstörungen stellen eine relevante Komplikation von Frakturen der Röhrenknochen dar.
- Bereits in der Frühphase einer Knochenheilungsstörung, optimal innerhalb der ersten 6 Monate nach Fraktur, ist die ESWT als eine vielversprechende und komplikationsarme Therapiemöglichkeit anzusehen.
- Mit steigendem Alter der Fraktur sinkt die Wahrscheinlichkeit für das Ansprechen auf die ESWT.
- Patienten mit einer Diastase ≥ 5 mm oder Instabilität der Osteosynthese sollten einem operativen Vorgehen zugeführt werden.
- Patienten mit Nikotinabusus, die sich einer ESWT unterziehen, sind zwingend auf eine Nikotinkarenz hinzuweisen.
- Im Vorfeld einer ESWT sollte immer eine Kostenübernahmeerklärung beim Kostenträger eingeholt werden.
